# Justification for requiring disclosure of diagnoses and prognoses to dying patients in saudi medical settings: a *Maqasid Al-Shariah*-based Islamic bioethics approach

**DOI:** 10.1186/s12910-022-00808-6

**Published:** 2022-07-13

**Authors:** Manal Z. Alfahmi

**Affiliations:** Bioethics; Executive Administration of Research and Innovation, King Abdullah Medical City, Makkah, Saudi Arabia

**Keywords:** Disclosure, Autonomy, Paternalism, Family dominance, Dying patients

## Abstract

**Background:**

In Saudi clinical settings, benevolent family care that reflects strongly held sociocultural values is commonly used to justify overriding respect for patient autonomy. Because the welfare of individuals is commonly regarded as inseparable from the welfare of their family as a whole, these values are widely believed to obligate the family to protect the welfare of its members by, for example, giving the family authority over what healthcare practitioners disclose to patients about their diagnoses and prognoses and preventing them from making informed decisions about their healthcare.

**Discussion:**

Family dominance over the healthcare decisions of competent patients is ethically problematic when the family prevent healthcare practitioners from disclosing diagnoses and prognoses to patients who have the capacity to consent and make decisions in their own best interests. Thus, the author holds that sociocultural values ought to be respected only when they do not prevent competent patients from knowing their diagnoses and prognoses or prevent them from making their own decisions.

**Conclusion:**

Healthcare practitioners should not allow patients’ families to control what can or cannot be disclosed to competent patients. This is particularly important when patients are approaching death so that they may address their material and spiritual wishes—among other needs—as they prepare for death. Justification for this position is drawn from the *Maqasid Al-Shariah-*based Islamic bioethics approach, from which it is possible to argue that the harm of withholding diagnoses and prognoses from patients who are imminently dying outweighs the potential benefits.

## Background

*During a night shift in a Saudi hospital, the author cared for an elderly female patient who was unaware that she was imminently dying from end-stage colorectal cancer. Her adult sons and daughters had insisted that healthcare providers should not disclose to their mother the truth about her health, which they had hidden from her for quite some time. Instead, they convinced her that the reason for her admission to the hospital was for a treatable colon infection and that she would be discharged within a few days. Despite her pain and suffering, she was kind and gracious to everyone until she suddenly collapsed due to shock caused by acute blood loss. She died in spite of strenuous efforts to resuscitate her. Her family was not blamed for hiding the truth from their deceased mother, and the healthcare providers who allowed this to occur were not blamed either. Both the family and healthcare providers continued their lives without thinking that they may have prevented this patient from handling her material and spiritual affairs, or even from saying goodbye to her loved ones, before dying.*

The patient described above experienced family dominance over her decisions that occurred regardless of her competency. Before discussing the moral justification for requiring the disclosure of diagnoses and prognoses to dying patients in Saudi medical settings, it is necessary to understand the sociocultural background of the Saudi Arabian, Middle Eastern community, where individuals with strong family bonds can rely on their family for continuous protection and support during good and bad times, and especially in times of need. The declarations of God in the *Quran* and of the Prophet Mohammed (peace be upon him (PBUHﷺ)) in the *Hadith* have always provided essential guidance when formulating Saudi Islamic laws and regulations in all sectors of life.

Arabian traditions and Islamic values both emphasise the importance of strengthening family bonds within the community, as strong family bonds are considered to form the essence of a healthy Islamic and Arabian community. Furthermore, by making family bonds essential, parents are rewarded by enjoying a relationship of lifelong mutual care and support with their children in both health and sickness. These traditions and values draw from the following verses:

And We have enjoined upon man, to his parents, good treatment. His mother carried him with hardship and gave birth to him with hardship, and his gestation and weaning [period] is thirty months. [He grows] until, when he reaches maturity and reaches [the age of] forty years, he says, “My Lord, enable me to be grateful for Your favour which You have bestowed upon me and upon my parents and to work righteousness of which You will approve and make righteous for me my offspring. Indeed, I have repented to You, and indeed, I am of the Muslims” [1].

That the Messenger of Allah (PBUHﷺ) said: “Indeed among the believers with the most complete faith is the one who is the best in conduct, and the most kind to his family” [2].

However, at times the support and care extended by families for their competent elderly parents and female family members can become dominating and paternalistic [3]. Although paternalism comes in different forms, the hard or strong types of paternalism^1^ are especially worrisome since they involve overriding the medical decisions of patients who have the capacity to consent or be adequately informed without controlling influences. The overriding of individual patient autonomy on the basis of sociocultural protective values is considered paternalistic when the patient has decisional capacity and expresses a desire to make their own decisions. Family care does not override respect for patient autonomy when patients authorise their family to make decisions on their behalf or when patients have medical reasons that can prevent them from making decisions in their own best interests.

Aljubran [4] notes that physicians in Saudi Arabia are expected to establish relationships with both the family and the patient and that most patients are unable to make health-related decisions without their family’s input. Families can also demand that a bad diagnosis be concealed from a patient, preventing the patient from making informed decisions about their future healthcare. Prioritising respect for patients’ sociocultural background instead of patient autonomy results in a range of harms, such as a lack of knowledge about one’s medical condition, violations of patient privacy and confidentiality, and a lack of opportunity to consent to medical interventions for oneself. It also makes it difficult to develop and sustain mutually honest and trusting doctor–patient relationships. Notably, in a qualitative study designed to assess patient attitudes towards the disclosure of cancer diagnoses and prognoses in Saudi Arabia, most of the patients interviewed preferred to know about their diagnoses, prognoses and treatment options. While the majority of patients also wanted their families to share in their decisions, they did not wish their healthcare practitioners to conceal important information from them in response to their families’ demands [5].^2^

Because she was unaware that she was imminently dying, the patient described in the case at the start of this section who died as a result of complications of colorectal cancer inspired the author to change the way patients like her are treated due to sociocultural protective influences. The author argues that there should always be a presumption in favour of clinicians telling the truth to patients, with any divergence from this standard requiring compelling justification. In the absence of medical justification for delaying or avoiding disclosure, giving the family authority to make decisions that they believe is in the patients’ best interests harms patients by preventing them from knowing their diagnoses and prognoses, which renders them unable to act in their own best interests.

Simply assuming that a patient lacks the ability to cope with the stress of knowing of a poor medical prognosis is not a legitimate reason for preventing disclosure. However, patients at high risk of exacerbating existing medical conditions are likely to benefit from delaying the disclosure of potentially distressing diagnoses and prognoses. Consider patients with a newly diagnosed disease that is serious and progressive but is not life threatening, as with degenerative diseases, like multiple sclerosis, Parkinson’s or Alzheimer’s disease. If these patients also have a co-existing heart disease or psychological condition, they may be vulnerable to harm from the stress of hearing about the new diagnosis, triggering further cardiac events or prompting self-harm or even suicide. Healthcare practitioners should weigh the benefits and harms of disclosure in such patients by assessing the stability of their existing medical conditions before new diagnoses of serious conditions are disclosed.

Nonetheless, the decision to withhold disclosures about the patients’ health should not apply to patients who are imminently dying, since from an Islamic perspective, the harm of withholding such information outweighs the potential benefits. Justification for this position is drawn from the *Maqasid Al-Shariah-*based Islamic bioethics approach. In this article, the author will draw on the *Maqasid Al-Shariah-*based Islamic bioethics approach to discuss how interpretations of what non-maleficence/*Darar* requires in Saudi healthcare settings to justify family paternalism.

The author argues that harm prevention should not be used to support culturally-based protective practices that prevent patients from acting in accordance with their own preferences unless there are health-related reasons for delaying disclosure. Thus, the author supports an understanding of non-maleficence/*Darar* that gives greater attention to what patients themselves regard as harmful.

## The *Maqasid Al-Shariah*-based Islamic bioethics approach

The primary sources of shariah law are the divine instructions of God in the *Holy Quran* and “reports about the sayings, actions and approvals of the prophet” (PBUHﷺ) in the *Hadith* [6, p. 479]. The principles of Islamic jurisprudence (or *usul al figh*) are derived from the foundational sources of shariah law by Muslim jurists [6]. These sources form the theoretical foundation of Islamic bioethics, as Muslim jurists refer to the literal interpretation of the sacred texts for legitimising responses to various ethical issues [7]. In addition to the *Quran* and the *Hadith,* other sources include *ijma* (“the consensus of the Muslim scholars regarding a particular issue at a particular time, which is considered to be legally binding”), *qiyas* (“analogical reasoning by Sunni Muslims”) and *aql* (“intellect by Shiite Muslims”) [6, p. 459]. There is no supreme juridical religious authority among Muslim communities and this, along with the diversity in the interpretations of the Islamic sacred texts among Muslim scholars, has caused contradictions in responses to certain bioethical issues [7]. To compact this ethical pluralism in dealing with emerging bioethical issues, the sacred sources of shariah law has “to be interpreted correctly” [7 p. 15].

When Muslim jurists face a medical ethical issue that is not clearly mentioned in the primary sources of shariah law and has no juristic precedent, they turn to the purposes of Islamic law, or *Maqasid Al-Shariah*, to tell right from wrong. However, they must avoid conflict with the spirit of the primary sources as they believe that these sources honour and govern their lives in health and sickness and are inseparable from Islamic morality [7]. These values draw from the following verse:

We have honoured the sons of Adam; provided them with transport on land and sea; given them for sustenance things good and pure; and conferred on them special favours, above a great part of our creation [8].

The basis of Islamic law aims to promote five purposes for individual life: “*din* as religion, *nafs* as life, *nasl* as progeny, *aql* as intellect, and *mal* as wealth” [6 p. 479]. Al-bar and Chamsi-Pasha [9] mention that one needs to explore the purposes of shariah law (*Maqasid*) to know about the philosophy of Islamic religion. In addition to *din* as faith, al-*nafs* as life, *al-aql* as mind, *al-nasl* as progeny, *al-mal* as property, they added *al-irdh* as honour [7 p. 50].

Thus, an action that would promote and preserve these five purposes (*maqasid*) is considered beneficial, while an action that would be to the detriment of all or any of these five purposes is considered harmful (it is worth noting that preventing harm is always required in Islam). All five components are linked such that by preserving the life of the individual, religion is also preserved; furthermore, by preserving individual lineages, minds and wealth, life is preserved [10]. Thus, Islamic medical ethics is linked to the five purposes of Islamic law, which take the holistic and comprehensive approach of *Maqasid Al-Shariah* as the basis for medical Islamic reasoning to promote patients’ wellbeing. Because wellbeing can be ensured by preserving patients’ religions, lives, lineages, minds and wealth, beneficial actions are those that preserve these purposes, and harmful actions are those that corrupt these purposes [6 p. 480–481].

The Islamic legal maxims, or *qawa id fiqhiyya,* are five abstract rules derived from the principles of jurisprudence (*usul al figh*) that are applicable to medical practice and constitute the basis for Islamic medical ethics. The first principle is *Qasd* (intention), wherein the moral evaluation of an act is dependent on the intention behind the action. This means that the morality of similar actions by healthcare practitioners shifts according to changes in their intentions [6]. For instance, while it is morally acceptable for a practitioner to give narcotics to a patient with terminal cancer if their intention is to relieve the patient’s pain and suffering, it is unacceptable if their intention is to hasten the patient’s death. In the Western tradition, this approach is the basis of the doctrine of double effect, which considers hastening a patient’s death to be morally acceptable if (amongst other conditions) it is a foreseen but unintended consequence of administering narcotics to relieve the patient’s suffering (respiratory depression that is likely to result in death is a foreseen side effect of giving morphine as a potent analgesic in palliative care settings) [6]. Furthermore, because each of the five purposes of shariah law in Islam is an end in itself, the maxim that the ends do not justify the means is also rooted in Islamic principles [10].

To clarify, a physician cannot justify the act of lying about their patient’s imminent death even if their intent is to promote the patient’s psychological wellbeing. This is because lying is prohibited in Islam, and the right action is the one that has the right intentions, motives and means [10]. While this often requires acts that minimise harm and maximise benefits, unlike the utilitarian approach, the intentions, motives and means must also remain ethical, as the best outcome alone will not justify the rightness of an action. Thus, healthcare practitioners are not permitted to use immoral methods, such as lying to avoid confronting patients with bad diagnoses or administering medically indicated narcotics with the intention of accelerating death, even if doing so can prevent patient suffering and achieve beneficial outcomes [10].

Cox and Fritz [11] maintain that lying, understood as “intentionally misleading” patients and telling them “false information” (p. 635) about their health, and withholding information, which they framed as intentionally “omitting” information, are morally equivalent if the physicians’ intentions and the consequences of each are the same. Moreover, lying to patients can be justified from a utilitarian perspective when it is likely to promote their wellbeing. For example, lying to patients with pre-existing heart disease about their new and serious diagnoses and prognoses may prevent them from experiencing additional stress or from developing new cardiac events that can worsen their overall medical condition as a result. In contrast, Beauchamp and Childress [12] claim that lying is more difficult to justify than staged disclosures or withheld information because lying can undermine trust between doctors and patients, which is the foundation of therapeutic relationships. They further argue that withholding information can be morally justified if the patient is suffering from other physical or psychological illnesses that prevent them from accepting the complete or partial truth about their bad diagnoses and prognoses. Thus, when diagnoses are not terminal and patients suffer from major depression or heart diseases that may prevent them from safely handling the truth, physicians can facilitate a staged or delayed disclosure with the help of psychologists, psychiatrists and/or social workers if these services are available or can be requested. However, lying is not justified under any circumstance, as lying can harm a physician’s relationship with their patient. This is especially true in cases where patients have a deadly disease (such as metastatic cancer). In such cases, concealing the truth about their diagnoses and prognoses may undermine patients’ trust in their physicians, especially if that concealment prevents them from managing their social, professional and financial affairs [13]. A common consequentialist criticism of including intentions when evaluating the morality of an act is that it is difficult, if not impossible, to verify intentions [12].

From an Islamic perspective, both the outcome and intention matter even if God is the only one who can know the intent behind certain actions^3^:

Allah will not call you to account for thoughtlessness in your oaths, but for the intention in your hearts; and He is Oft-forgiving, Most Forbearing [14].

Your Lord is most knowing of what is within yourselves. If you should be righteous [in intention]—then indeed He is ever, to the often returning [to Him], Forgiving [15].

The second principle is *Yaqin* (certainty), which according to Mustafa [6] is consistent with what we refer to today as “evidence-based medicine”:

It acknowledges that most medical decisions are relative and based on probability. Although *yaqın̄* (certainty) is theoretically the ideal, sound medical judgement as a minimum should be based on *ghalabat alzạnn* (predominant conjecture), which is superior to *zạnn* (mild inclination) or *shakk* (doubt). A further subprinciple in this area is that certainty is not removed by doubt (*al yaqın̄ la yazūlu bi al shakk*). An existing established truth or known assertion is only to be modified or disregarded with compelling evidence, and cannot be removed by an uncertainty or doubt [6 p. 480].

In other words, we should only revise our current understanding of particular issues if there is strong evidence and/or reason for doing so.

The third principle is *Darar* (injury), which justifies medical interventions that are necessary to relieve injury and prevent harm to the patient. The minimisation of harm via medical interventions takes priority over the pursuit of benefits (the justification for this is derived from the primary sources of shariah law). Moreover, if two harms coexist, then the lesser of two harms must be selected to prevent the greater harm [6]. For example, in the case of contagious diseases, lessening the harm to the public is prioritised over lessening the harm to patient confidentiality when mandatory reporting is required.

The fourth principle is *Darura* (necessity), which permits prohibited actions only when it is necessary to save a human life or prevent a disability when there is no other option and asserts that prohibition should resume once those actions are no longer necessary [6]. One example of this is using alcohol to sedate a severely injured patient when there is no anaesthetic available during an operation.

The fifth principle is *Urf* (custom), which states that local customs should be respected as long as they do not violate shariah law [6]. This means that an action that follows customs can be right as long as these customs are not against Islam or oppose Islamic principles [10]. For example, if customs allow a father to only distribute his wealth amongst his sons but deprive his daughters of inheritance, it is impermissible to do so because, according to Islamic law, although males should receive a greater proportion of their parents’ wealth than females, females should still be granted an inheritance^4^:

If there are both brothers and sisters, the male will have the share of two females. Allah makes clear to you [His law], lest you go astray. And Allah is Knowing of all things [16].

In summary, the *Maqasid Al-Shariah*-based Islamic bioethics approach applies Islamic principles to biomedicine so that the knowledge derived from biomedical research and practice can be employed in ways that are consistent with the values of Islam [10]. Thus, the demands of both physical and spiritual health need to be balanced in order to bring about “the needs of humankind to the maximum” [10 p. 338].

This practical approach can be flexibly applied to different times and situations for the evaluation of contemporary ethical issues. It also seeks to preserve the interests (*maslahah*) of both the individual and the public while preventing harm (*mafsadah*) to the five living purposes. Although each of the five purposes functions as an end and of itself, the *Maqasid Al-Shariah*-based approach provides guidance to resolve conflicts between any of these purposes by selecting the purpose that is superior in representing the greatest interest (or *maslahah)* that must be preserved, while setting aside the purpose that has the most detrimental interest (or *mafsadah*). As Ibrahim et al. [10] state, “Preservation of the religion and life is given priority over the preservation of wealth. This priority is because wealth can be replaced while the loss of life and religion is permanent in nature” [10 p. 339]. When deciding which purpose is superior in representing the greatest interest (or *maslahah*) and which constitutes the purpose that has the most detrimental interest (or *mafsadah*), the potential consequences of any proposed actions are evaluated for their degree of certainty (whether it is certain, assumed or doubted to happen) [10]. For example, if a pregnant mother’s life is put at risk by continuing her pregnancy, termination of the pregnancy is permissible if doing so could save her life.

Furthermore, the potential consequences of any proposed actions are evaluated for their inclusivity (whether they involve the public interest or only individual interest) [10]. For instance, the implication of putting patients infected with a contagious disease in quarantine can certainly enhance public interest in preventing the spread of the disease.

In addition, ethical issues must be evaluated in relation to their implications for the three following factors: intention, method and outcome on the five purposes of religion, lives, lineage, minds and wealth. These implications should primarily be positive in terms of promoting the interest or *maslahah* rather than negative in terms of causing harm or *mafsadah*. For instance, the implication of not disclosing bad prognoses to patients who are imminently dying can certainly affect the public interest when patients are prevented from handling their material and financial affairs before dying. Telling patients the truth about their imminent death might serve the public interest through the just dissemination of monetary resources (e.g., paying or directing the family to pay the patient’s debt).

Islam values individual autonomy but also “the potentially competing values of family, society” and “the consideration of public interest” [6 p. 482]. Therefore, the principle of respect for patient autonomy, which requires healthcare workers not to impede patients but rather to facilitate decisions that are in their best interests and in accordance with their beliefs (as long as their choices do not contradict Islamic principles), is rooted in Islam. As an example, although this principle can involve honouring a patient’s choice to intentionally end his/her life to relieve pain and suffering, this contradicts Islamic principles since euthanasia is forbidden in Islam. This is because pain and suffering are considered to be part of the lived journey and Muslims believe that God will reward them for their patience in the afterlife:

And do not kill the soul which Allah has forbidden, except by right. And whoever is killed unjustly—We have given his heir authority, but let him not exceed limits in [the matter of] taking life. Indeed, he has been supported [by the law] [17].

Indeed, the patient will be given their reward without account [18].

## The moral justification for requiring disclosure to dying patients from an Islamic perspective

In this section, the author provides the moral justification for requiring disclosure to dying patients from an Islamic perspective referring to the shariah law and the five purposes of Islamic law as the foundation of Islamic bioethics and Islamic medical ethics. As mentioned earlier in this article, healthcare providers might seek to justify withholding the truth from an imminently dying patient to avoid harming them psychologically, even if this conflicts with respecting the patient’s autonomy. However, given that patients are likely to have strong preferences for making informed choices about their clinical, spiritual and material needs when death is near, it is more harmful to not consider patient preferences than to risk other harms, even if these may hasten the patient’s health. Additionally, healthcare practitioners must ensure that concerns about patient harm are not used to justify families overriding patients’ autonomy due to sociocultural protective influences or without a medical justification for delaying disclosure. This requires an understanding of *Darar* or patient harm that gives greater attention to patients’ preferences when identifying their best interests in medical decision-making, particularly for patients who are imminently dying. The reason for this is at the end of life, the long-lasting harm of not considering the dying patient’s preferences to know about their imminent death would outweigh any short-lasting benefit that maintains their psychological health because concealing the truth from patients about their imminent death can result in permanent harm when those patients are prevented from handling their material and spiritual affairs before dying.

Muslims are encouraged to gain knowledge that will help them make “reasoned decisions” that enable them to choose the right path according to the divine rules [6 p. 482]. Rattani and Hyder [19] observe that despite claims that Islam undermines human will and rights, there is strong evidence in the *Holy Quran* that various prophets made autonomous decisions that either drove them away from or in the direction of the right path. Verse 2:53 of the *Al-Qur’an* states: “Then We gave Musa the Book and the Criterion (of right and wrong) so that you find the right path” [20]. This verse clearly shows that one needs to be informed about their circumstances so they can choose the right path, and patients who are imminently dying need to know the truth about their imminent death to find the right path in handling their life and death issues before it is too late for them to do so.

Thus, the disclosure of medical information to patients is necessary for them to be informed and knowledgeable of their circumstances. This is consistent with the five purposes of shariah law, as consenting to medical decisions that promote one’s their interests while meeting the requirements of the five purposes makes them more capable of choosing the right path.

Support for this approach can also be found in Alzahrani et al.’s [21] study of the attitudes of cancer patients and their families towards the disclosure of cancer diagnoses. The study surveyed 304 cancer patients and 277 family members at the oncology outpatient clinic and inpatient oncology department of King Abdullah Medical City, one of the largest cancer units in Saudi Arabia.^5^ The authors concluded that while most patients (83.6%) preferred to be informed about the details of their diagnoses or stage of cancer (an indicator of their prognoses), only 59.9% of families agreed that patients needed to be informed about such details.^6^ The attitude of these patients was more accepting of their diagnosis and hopeful for a cure. In comparison, the families preferred limiting disclosure to the patient out of fear that any negative emotional response could impede their recovery from cancer. The three main factors associated with the patients’ acceptance of their cancer diagnoses included religious beliefs, effective communication in the doctor–patient relationship and the support they received from their family and friends.

Zekri and Karim [13] hold that physicians are obligated to make families aware of the positive implications of informing patients about their diagnoses and prognoses. A patient’s awareness of their medical condition is important because it can empower them to manage their social, professional and financial affairs, especially if they have a terminal disease, such as metastatic cancer. Under circumstances like these, concealing a bad diagnosis or hopeless prognosis might prevent the patient from achieving these personal goals. More generally, involving patients in decisions is thought to improve their quality of life by increasing their acceptance of medical conditions and helping to ensure their compliance with management plans [4].

Bou Khalil [22] discusses a study from Turkey^7^ that supports the concern that telling the truth to cancer patients may result in negative consequences that increase their stress and cause them to suffer from psychiatric problems that rob them of the hope for a cure, ultimately harming them more than the disease itself. Nevertheless, Husson et al.’s systematic review of studies that examine the relationship between information disclosure and health-related quality of life, anxiety and depression among cancer survivors concluded that patient-reported anxiety levels were higher in uninformed patients, and that the disclosure of cancer diagnoses improved patients’ quality of life by increasing their compliance and management of symptoms (as cited in [21]).

Islam emphasises that it is important for an individual to write a will before death. A person’s will should not only address the matter of how they would like their wealth and possessions to be distributed to their relatives but, most importantly, should also include the debts they owe to other people since debts must be paid before their death (or the family should at least be informed so they can make arrangements to pay the debt in full when it is due). Thus, telling patients the truth about their imminent death is essential because only then can they have the opportunity to manage their material and financial affairs. This also promotes the interests of the public, which is essential in Islam. If patients are not told about their imminent deaths, they unintentionally risk mistreating the people to whom they owe debts:

Prescribed for you when death approaches [any] one of you if he leaves wealth [is that he should make] a bequest for the parents and near relatives according to what is acceptable—a duty upon the righteous [24].

The Prophet (PBUHﷺ) said: “After the grave sins which Allah has prohibited the greatest sin is that a man dies while he has debt due from him and does not leave anything to pay it off, and meets Him with it” [25].

Telling dying patients about their imminent death also gives them the opportunity to genuinely repent for their sins in the hope of being saved, forgiven and included in God’s mercy before death:

The Prophet (PBUHﷺ) said, “Allah accepts a slave’s repentance as long as the latter is not on his death bed (that is, before the soul of the dying person reaches the throat)” [2].

Thus, from an Islamic perspective, telling the truth to imminently dying patients about their diagnoses and prognoses is required. This is because the positive implications of telling the truth, which promote the purposes of faith and religion by enabling the patient to genuinely repent and serve the public interest through the just dissemination of monetary resources (paying or directing the family to pay the patient’s debt), outweigh the negative implications that arise from attempts to preserve the patient’s psychological health and state of mind. In other words, because their death is already certain, the long-lasting benefits of paying a patient’s debts and strengthening their faith are superior to preventing the temporary harms of exacerbating a patient’s heart disease or psychological condition. Withholding the bad diagnosis and prognosis will not save their physical life but will prevent them from having an opportunity to save their spiritual life. The author holds that any proposed action should be evaluated from what is beneficial or harmful and what is permanent or temporary. Thus, preventing long-lasting harm to the dying patient’s material and spiritual affairs must be prioritised over promoting short-term benefits to the patient’s physical and psychological health. However, for patients who are not imminently dying, harm caused by information about those patients’ physical and psychological health might be more long lasting, and disclosure needs to be delayed till their condition is stable enough for them to be able to cope with the truth.

In summary, the author maintains that failing to tell the complete truth about medical diagnoses and prognoses to patients who are not imminently dying is not as ethically problematic as deliberately lying to them (e.g., when there is a high risk of exacerbating existing conditions). Nonetheless, failing to disclose the truth to patients about their imminent death can be as immoral as lying to them, as patients will be harmed either way. A physician cannot justify the act of lying about their patient’s imminent death even if their intent is to promote the patient’s psychological wellbeing. This is because lying is prohibited in Islam, and the right action is the one that has the right intentions, motives and means [10]. Put differently, not telling a patient the truth is similar to lying to them because the patient is prevented from managing their material and spiritual interests before death in both cases.

The Islamic perspective requires the disclosure of the truth to patients about their imminent death so that they can take care of their material and spiritual interests. The medical team’s prediction of the patient’s imminent death must be based on valid and accurate investigations and medical assessments before the news of the fatal prognosis is broken to the patient. Neither medical nor sociocultural reasons should prevent the patient from knowing this information.

Nonetheless, the author argues that physicians should not share their precise predictions for when the patient might die since patients may prefer not knowing this information. If the patient insists on knowing, they should be made aware that the timeframe is only a prediction and that physicians cannot be certain. In particular, Muslims believe that people can never know when or where they are going to die, as no one can be certain since their lives are in God’s hands:

Indeed, Allah [alone] has knowledge of the Hour and sends down the rain and knows what is in the wombs. And no soul perceives what it will earn tomorrow, and no soul perceives in what land it will die. Indeed, Allah is Knowing and Acquainted [26].

## Conclusion

The Islamic principle of *Darar* (which is equivalent to the principle of non-maleficence) is often used inappropriately by healthcare practitioners to justify a family’s right to override patient autonomy for culturally-based protective reasons. The author argued that the avoidance of harm for sociocultural protective reasons should not be used to prevent patients from acting in accordance with their own preferences unless there are compelling medical reasons for delaying disclosure. This was supported by placing more emphasis on patient preferences when considering patient harm; i.e., when the harm of preventing patients from knowing their diagnoses and prognoses outweighs the benefit.

The author argued that telling patients who are imminently dying the truth about their imminent death is essential and mandatory. This was justified from an Islamic perspective based on the importance of patients being informed of their imminent death so that they have an opportunity to manage their material, spiritual and financial affairs. However, if a patient is not facing imminent death and has pre-existing conditions that could worsen from the disclosure of diagnoses and prognoses, risk assessments of their existing condition may justify physicians delaying the disclosure of medical information to avoid exacerbating their condition. Nonetheless, lying to patients or preventing disclosure cannot be ethically justified under any circumstances.

## Data Availability

Not applicable.

## Footnotes

1 Soft or weak forms of paternalism involve making medical decisions on the behalf of patients who are somehow defective in their decision-making capacities (e.g., they may be ignorant of relevant facts or insist on pursuing means that enable them to achieve their ends).

2 Though the study was conducted with a small sample of 114 cancer patients in a single hospital located in the Eastern Saudi province, the participants were from different Saudi regions: 61% from the Eastern province, 21% from the Southern province, 8% from the Central province, 5% from the Northern province and 4% from the Western province. At least 59% of the participants were female, 41% were male and their ages ranged from 18 to 85 years old. At least 59% of the participants were illiterate and the participants’ educational experiences ranged from primary (10%), intermediate (10%) and secondary school (10%) to university degrees (14%). Thus, this study, which was conducted between January 2002 and December 2005, was representative of the Saudi population [5].

3 From a practical perspective in healthcare settings, this might not be a convincing justification for the appropriate action. However, in Islam, there is a strong emphasis on using good intentions to achieve good outcomes and to make certain actions right.

4 In Islam, while fathers, husbands and sons are obligated to support their daughters, wives or mothers with their living expenses even if those women have their own money, women are not obligated to support anyone with their money unless they desire to do so.

5 The patients were predominantly female (62.4%), middle-aged (a mean age of 48.3 years) and unemployed (79.02%). In addition, 50.2% of the patients had not completed high school. Most family members were male (53.1%), young (a mean age of 36.5 years), and most of them were either the sons or daughters (41.8%) or husbands or wives (18.1%) of the patients. The remaining family members were the patients’ mothers, fathers and brothers or sisters (12.4%, 5.6% and 11.9%, respectively).

6 A P-value of less than (< 0.001) indicates a statistically significant difference.

7 Atesci et al. [23] found that 28.7% of cancer patients have a DSM-IVAxis I diagnoses, which shows a positive correlation between anxiety and depression scores with disclosures of cancer diagnoses and prognoses to patients (*p* = 0.001). Such an association may be related to physicians’ insufficient communication skills during the disclosure of cancer diagnoses and prognoses (cited in [22]).

## References

[1] Al-Qur’an, *Surah Al-Ahqaf* 46:15. https://quran.com/46/15.

[2] *Hadith, Jami` at-Tirmidhi* 1:18. http://sunnah.com/riyadussaliheen/1/18.

[3] Dworkin G. Paternalism. In: Zalta, EN, editor. Stanford Encyclopedia of Philosophy. Stanford University. 2020. https://plato.stanford.edu/archives/sum2020/entries/paternalism/.

[4] Aljubran AH (2010). The attitude towards disclosure of bad news to cancer patients in Saudi Arabia. Ann Saudi Med.

[5] Al-Amri AM (2009). Cancer patients’ desire for information: a study in a teaching hospital in Saudi Arabia. East Mediterr Health J.

[6] Mustafa Y (2014). Islam and the four principles of medical ethics. J Med Ethics.

[7] Atighetchi D (2007). Islamic bioethics: problems and perspectives.

[8] *Al-Qur’an, Surah Al-Israa* 17:70. https://quran.com/17/70.

[9] Al Al-Bar MA, Chamsi-Pasha H (2015). Contemporary bioethics: Islamic perspective.

[10] Ibrahim AH, Rahman NNA, Saifuddeen SM, Baharuddin M (2019). Maqasid al-shariah based Islamic bioethics: a comprehensive approach. J Bioethic Inq.

[11] Cox CL, Fritz Z (2016). Should non-disclosures be considered as morally equivalent to lies within the doctor–patient relationship?. J Med Ethics.

[12] Beauchamp TL, Childress JF (2019). Principles of biomedical ethics.

[13] Zekri J, Karim SM (2016). Breaking cancer bad news to patients with cancer: a comprehensive perspective of patients, their relatives, and the public—example from a Middle Eastern country. J Glob Oncol.

[14] *Al-Qur’an*, *Surah Al-Baqara* 2:225. https://quran.com/2/225.

[15] *Al-Qur’an*, *Surah Al-Israa* 17:25. https://quran.com/17/25.

[16] *Al-Qur’an*, *Surah An-Nisa* 4:176. https://quran.com/4/176.

[17] *Al-Qur’an*, *Surah Al-Israa* 17:33. https://quran.com/17/33.

[18] *Al-Qur’an*, *Surah Az-Zumar* 39:10. https://quran.com/39/10.

[19] Rattani A, Hyder AA (2017). Developing an Islamic research ethics framework. J Relig Health.

[20] *Al-Qur’an, Surah Al-Bagara* 2:53. https://quran.com/2/53.

[21] Alzahrani AS, Alqahtani A, Alhazmi M (2018). Attitudes of cancer patients and their families toward disclosure of cancer diagnosis in Saudi Arabia: a Middle Eastern population example. Patient Prefer Adher.

[22] Bou Khalil R (2012). Attitudes, beliefs and perceptions regarding truth disclosure of cancer-related information in the Middle East: a review. Palliat Support Care.

[23] Atesci FC, Baltalarli B, Oguzhanoglu NK, Karadag F, Ozdel O, Karagoz N (2004). Psychiatric morbidity among cancer patients and awareness of illness. Support Care Cancer.

[24] *Al-Qur’an. Surah Al-Bagara* 2:180. https://quran.com/2/180.

[25] *Hadith, Sunan Abi Dawud* 23:17. http://sunnah.com/abudawud/23/17.

[26] *Al-Qur’an, Surah Luqman* 31:34. https://quran.com/31/34.

